# Nanovesicles drive a tunable dynamical arrest of microparticles†

**DOI:** 10.1039/d1ra04252a

**Published:** 2021-07-09

**Authors:** Francisco Javier Guevara-Pantoja, J. C. Ruiz-Suárez

**Affiliations:** CINVESTAV-Monterrey, PIIT Autopista Nueva al Aeropuerto Km. 9.5 Apodaca Nuevo León 66600 Mexico jcrs.mty@gmail.com

## Abstract

Vitrification in a dilute colloidal system needs an asymmetric particle composition (a mixture of nano and micro colloids) to materialize. The volume fraction of the large particles increases (up to ≈0.58) driven by depletion forces produced by the smaller colloids. Such entropic forces are short-ranged and attractive. We found a different type of dynamical arrest in an extremely dilute asymmetric mixture of nanovesicles and polystyrene microparticles, where energy, instead of entropy, is the main protagonist to drive the arrest. Furthermore, when the vesicles go through the gel-fluid phase transition, the mean square displacements of the microparticles suffer a sudden splitting indicating a viscous jump. If the vesicles are doped with negatively charged lipids, particles and vesicles repel each other and the rheology of the mixture becomes athermal and Newtonian. Our findings are important to understand caging phenomena in biological systems, where diverse electrostatic distributions are present.

## Abstract 2

A dynamical arrest in an extremely dilute asymmetric mixture of nanovesicles and polystyrene microparticles was discovered, where energy, instead of entropy, is the main mechanism to produce it.

## Graphical abstract

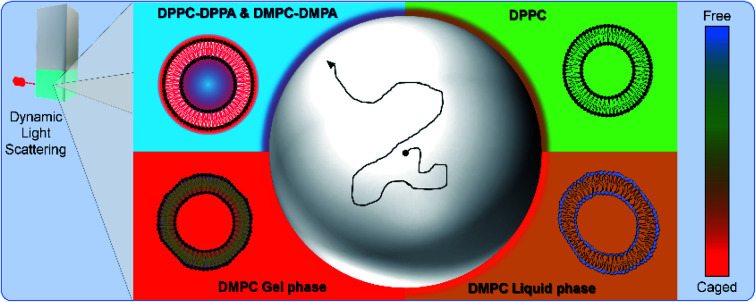

## 1 Introduction

Research to understand the physical subtleties behind the formation of vitrified states, is still very active in the soft matter scientific community.^1–5^ Although it is hard to describe it in a few words, a succinct description of a vitrification process should include the fact that when a colloidal system vitrifies, either by adding mass, reducing volume or temperature, or by shear, there appears a prominent dynamical arrest of the colloids. This phenomenon is observed not only in hard-sphere mixtures,^1,2,5,6^ but in soft ones.^3,7^

An interesting manifestation of the glass formation phenomenon arises in asymmetric systems (mixtures of small and large colloids),^8^ where competing interactions known as short-range attraction and long-range repulsion (SALR) interactions, may drive equilibrium cluster phases, equilibrium gels, and Wigner glass of clusters.^5^ In dilute conditions, clusters nucleate by depletion or entropic forces (short-range) and stabilize by electrostatic repulsion (long-range). At higher concentrations we may observe merging of such clusters into a percolating gel or more robust ones that keep their integrity (Wigner glass). In soft systems, a good model for the smaller colloids is provided by star polymers.^3,9^

The mean square displacements (MSD) of the larger colloids, or tracers, give us information about their dynamics in a glass forming state: at very low lag times the MSD follow a *t*^2^ diffusion due to rattling within cages; at intermediate lag times, the MSD become linear in time (*t*),^5^ meaning that tracers scape from the cages formed by neighbours and diffuse freely. If one keeps inspecting the MSD for larger lag times, subdiffusion might be observed, reflecting the impossibility to scape from the cages due to a crowded environment around the particles.^10^ As far as we know, subdiffusion has not been observed in asymmetric hard–soft mixtures.

We studied very dilute asymmetric mixtures made of hard (polystyrene ∼ 1 μm) and soft (vesicles ∼ 20 nm) colloids. Unexpectedly, we discovered a dynamical arrest with two novel features: depletion forces do not participate and a subdiffusion process enters into the scene. In principle, such a mixture would have an ideal Newtonian behavior, for at the dilute conditions of the experiments, there is no crowding to hinder the free diffusion of the tracers. Nevertheless, we observed that tracers are caged not by like particles as in other glass forming systems, but by a sudden augment of vesicles attracted by electrostatic forces. Moreover, such response is thermally tunable due to the gel-fluid transition of the nanovesicles. If electrostatic repulsion between microparticles and vesicles is induced by doping the vesicles with charged lipids, the suspension recovers the purely viscous behaviour typical of dilute suspensions.

**Fig. 1:**
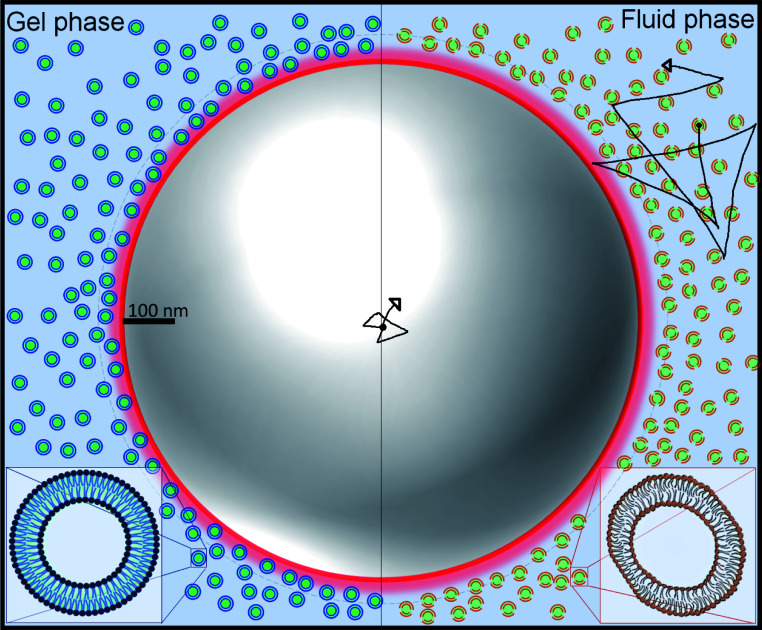
Graphical representation of SUVs and a tracer. The left (right) part of the image represents the gel (fluid) phase of the vesicles, respectively. In the gel (fluid) phase, the vesicles are rigid (soft and deformable). Note the much larger size of the tracer particle compared to the small size of vesicles.

The suspensions were prepared by suspending in water small unilamellar vesicles (SUVs) made of 1,2-dipalmitoyl-*sn*-glycero-3-phosphocholine (DPPC) and 1,2-dimyristoyl-*sn*-glycero-3-phosphocholine (DMPC). The lipid vesicles, which have an average bilayer thickness of 4 nm,^11^ self assemble due to the amphiphilic nature of lipids, where the tails of two contiguous lipids remain near each other and their heads in contact with water. We then introduce micrometric tracer particles in the SUVs suspensions. Since SUVs barely scatter light (due to their very small size) we follow the tracers by measuring the light they scatter. We illustrate this system with the help of Fig. 1, where the scales of the SUVs and a tracer particle are roughly indicated. Fig. 1 also highlights the difference in the morphology of the SUVs before and after the thermal transition.

## 2 Experimental and methods

### 2.1 Sample preparation

1,2-Dipalmitoyl-*sn*-glycero-3-phosphocholine (DPPC), 1,2-dimyristoyl-*sn*-glycero-3-phosphocholine (DMPC), 1,2-dipalmitoyl-*sn*-glycero-3-phosphate (sodium salt) (DPPA) and 1,2-dimyristoyl-*sn*-glycero-3-phosphate (sodium salt) (DMPA) all lipids with purity >99% were obtained from Avanti Polar Lipids (Alabaster, Alabama). Chloroform (purity >99.8%), methanol (purity >99.8%) and dichloromethane (purity >99.5%) were purchased from Sigma Aldrich (Toluca, Mexico). 0.994 μm polystyrene microspheres (4009A) from Duke Standards (Monterrey, Mexico).

DPPC powder was hydrated with deionised water (18.2 MΩ or 0.1 mS cm^−1^) while stirring at 300 rpm with a magnetic bar at 50 °C. This process creates giant multilamellar vesicles. The small unilamellar vesicles (SUV) were obtained by bath sonication well above the transition temperature, *T*_m_ (65 °C). After at least 4 hours of sonication per sample, the size distribution was measured. The sample was sonicated again if the SUVs did not have the desired size distribution, see Fig. 1 in the ESI.† DMPC SUVs were elaborated following the same protocol. However, in this case, the sonication bath temperature was set at 50 °C. DPPC-DPPA and DMPC-DMPA (95–5 w/w) SUVs were prepared by solubilizing DPPC (DMPC) and DPPA (DMPA) in a mixture of chloroform, methanol and dichloromethane (2 : 1 : 1). Subsequently, they were evaporated for 20 hours with magnetic stirring at 50 °C (65 °C) in an air extractor cabin. As a result, we obtained DPPC-DPPA (DMPC-DMPA) powder and thus followed the same protocol used for the DPPC (DMPC) SUVs.

Polystyrene microspheres (0.994 μm) were added to the SUVs suspensions just before the experiment. 12 μl of the purchased particles (suspended in water at 1% v/v) for each mg of lipids was employed. The scattering intensity generated by the polystyrene particles is sufficiently large to screen the scattering intensity produced by the nanovesicles. The volume fraction of liposomes was calculated (see ESI†) to be 0.019 for the 15 mg ml^−1^ concentration used in the experiments (it was estimated with previews data^11,12^ summarized in Table 1 in the ESI).† Moreover, as suggested by an earlier work,^13^ size measurements were performed before and after the microrheology experiment in order to check for particle aggregation. No liposome aggregation was found during the experiments, see ESI.† For the reported data it was found that 99% of the volume in 12 samples contained vesicles with an average diameter of 23.33 nm with a standard deviation of 9.36 nm.

### 2.2 Dynamic light scattering

Dynamic light scattering experiments to measure MSD were performed using a Malvern Zetasizer Nano ZSP. All diffusion experiments were carried out using the same settings: 0.85 mm from the 0.5 ml cuvette wall, 30 runs with a duration of 30 seconds, automatic attenuator selector and 173° backscattering light. Three different experiments for each condition were performed and averaged. See analysis in the ESI.†

### 2.3 Zeta potential

In addition to the MSD measurements, we also obtained the zeta potential (*ζ*) of tracers and vesicles. The sample cuvettes of 0.5 ml were degassed (at 635 mmHg) for 5 minutes and sealed to avoid evaporation. 100 runs of 30 seconds and 3 measurements per sample were performed. A stabilization time of 10 minutes was given after each temperature change in the zeta/temperature experiment. We obtained the values of zeta potential using a phase analysis light scattering (PALS)^13^ technique and the electrophoretic mobility (*μ*) based on the Smoluchovski model (*μ* = *εζ*/*ν*), where *ε* is the dielectric constant and *ν* the viscosity of the medium.

### 2.4 Calorimetry

Heat capacity profiles were obtained by Differential Scanning Calorimetry (DSC). The lipid concentration was 3.5 mg ml^−1^; a heating rate of 1 °C min^−1^, and a pressure of 3 atm were used. The lowest and highest temperatures were selected according to the lipid main phase transition temperature reported in the literature for LUV's.^14,15^ The samples were degassed 10 min at 635 mmHg of vacuum pressure and 25 °C before they were loaded in the calorimeter (Microcalorimeter, NanoDSC, TA Instruments). Data were analysed using the software provided with the instrument.

## 3 Results

**Fig. 2:**
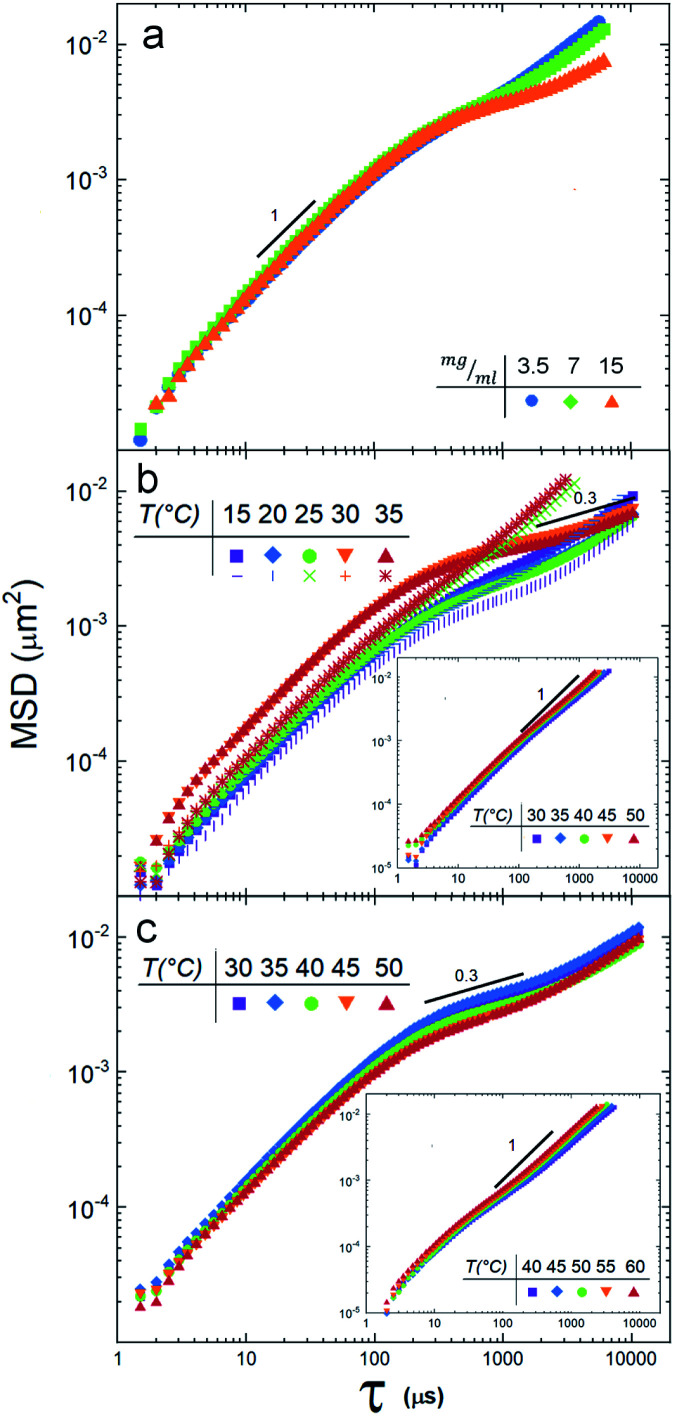
(a) Mean squared displacements of tracer particles in a DPPC suspension at 25 °C for three concentrations, 3.5 mg ml^−1^ (blue circles), 7 mg ml^−1^ (green squares) and 15 mg ml^−1^ (orange triangles). (b) Mean squared displacements of particles in the DMPC suspension at 5 temperatures, heating from 15 to 35 °C (solid symbols). When the suspension is cooled, from 35 °C to 15 °C, the response is different for temperatures higher than *T*_m_. Note that the diffusion anomaly is not seen (the MSD are linear). See discussion in the text. Inset: MSD when DMPA is added to DMPC vesicles. (c) Mean squared displacements of tracers in the SUVs suspension of DPPC at 5 temperatures. Inset: 5% of DPPA lipids were added to the DPPC vesicles.

Fig. 2a shows the MSD of the tracers for three different vesicle concentrations: 3.5 mg ml^−1^, 7 mg ml^−1^ and 15 mg ml^−1^. Note that at lag times around 300 μs, the MSD change slope, indicating the beginning of an anomalous sub-diffusive regime. Since no mesh or elastic network exists in the medium to hinder the diffusion of the particles, the reason to observe a deviation must be in the nature of the particle/vesicle interactions, see Discussion section. It is important to remark that the sample with the highest concentration (15 mg ml^−1^) deviates more notoriously, so we decided to use this concentration in the next experiments.

Next, we measured the thermotropic responses of the SUVs to find their phase transitions needed to determine the temperature ranges in which we will make the DLS experiments.

**Fig. 3:**
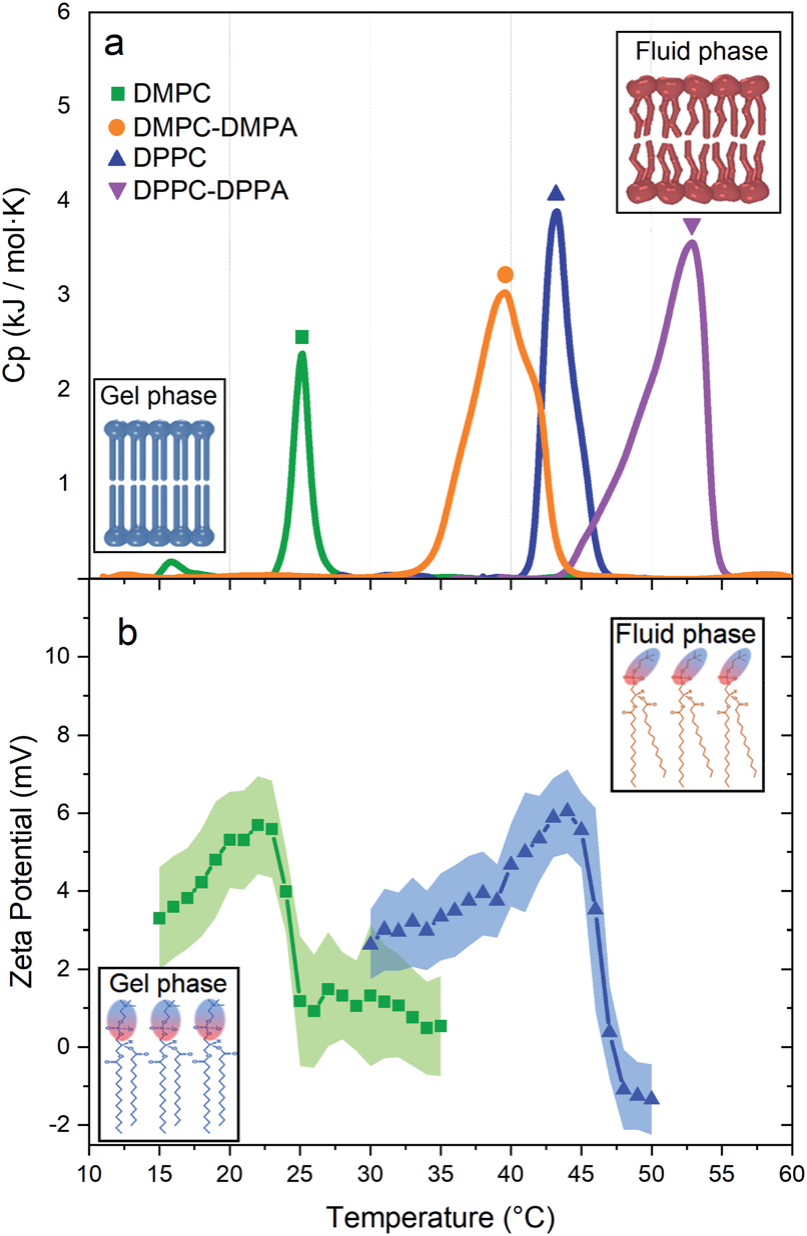
(a) Thermotropic profiles of DMPC (*T*_m_ at 25.16 °C), DMPC-DMPA (*T*_m_ at 39.6 °C), DPPC (*T*_m_ at 43.25 °C), and DPPC-DPPA (*T*_m_ at 52.85 °C) vesicles. Insets: structure of the lipid bilayer before and after the phase transition. (b) Zeta potentials *vs. T* for DMPC and DPPC vesicles. Insets: polar heads of lipids showing their orientation in the gel and fluid phase.

Fig. 3a shows the thermotropic profiles and transition temperatures for DMPC, DMPC-DMPA, DPPC, and DPPC-DPPA vesicles. We would like to remark that despite previous coarse-grained molecular dynamic simulations, indicating that SUVs did not manifest phase transitions,^16^ our measurements probe that such transitions do exist. Interestingly, the obtained transition temperatures are a bit higher compared to temperatures reported for large vesicles.^14^ Note also that only DMPC vesicles present a pre-transition peak (around 16 °C), which according to previous studies^17–19^ is related to a rippling phase highly dependent on the curvature and lateral stress of the bilayers, see Table 1 in the ESI.† We assume that DPPC SUVs do not show a pre-transition peak because, due to the larger hydro-carbonated tails, there is a higher lateral stress. The same occurs with the DMPC-DMPA and DPPC-DPPA SUVs.

Fig. 2b depicts the tracer's MSD obtained for the DMPC vesicle mixture at temperatures below and above the phase transition temperature (25.16 °C). We note a clear difference between the gel phase (15 °C and 20 °C) and the fluid phase (30 °C, 35 °C): *i.e.* the MSD splits when the temperature crosses *T*_m_. These measurements reveal also that the suspension is non-Newtonian, since there is a regime where, for lag times larger than ≈200 μs, the diffusion is anomalous, see the Discussion section.

A different scenario appears if the DMPC vesicles are doped with (5%) of charged DMPA lipids^20^. In this case, the MSD are fully linear (see inset in Fig. 2b).

SUVs constituted by larger hydrocarbonated tails, but still with zwitterionic polar heads (DPPC), are now investigated. Fig. 2c depicts the MSD of particle tracers at temperatures below and above the SUVs phase transition temperature *T*_m_ (43.25 °C, see Fig. 3a). While the deviations for large lag times are clearly noticed, the MSD split in a lesser extent during the phase transition. In a similar way DMPC SUVs were electrically charged by 5% of DMPA, DPPC SUVs were doped by 5% of DPPA. Note that the transition temperature shifted to 39 °C (Fig. 3a) and the MSD linearized and collapsed, see inset in Fig. 2c.

## 4 Discussion

We have shown that suspensions constituted by a mixture of polystyrene microparticles and nanovesicles give rise to interesting diffusive responses. If the vesicles are made of pure zwitterionic lipids, the polystyrene particles diffuse freely at short times, thereafter they sub-diffuse, see Fig. 2b and c. We also found that the MSD split when the vesicles pass through their phase transitions. These intriguing behaviors (sub-diffusion and splitting) cannot be explained by invoking short-range depletion forces. The mixture is very dilute and entropic forces must be negligible, furthermore, depletion forces would not change during gel-fluid transitions. Since the only clue we have to explain the anomalous diffusions shown in Fig. 2b and c is their evident evanescence after doping the SUVs with negative lipids (DMPA and DDPA), the next obvious step to elucidate the observed phenomenon is the careful inspection of the electrical properties of both the microparticles and SUVs. Hence, their zeta potentials were measured as a function of temperature. In Fig. 3b we show the zeta potentials of DMPC and DPPC SUVs. Surprisingly, despite the fact the lipids forming the vesicles are zwitterionic and for such reason the latter should bear no electric charge and therefore being in the isoelectric point (zero zeta potential), we found that when they are in the gel phase (below the transition temperature), they have positive zeta potentials. The origin of these potentials should be in the ordered structures of the lipids: their phosphate groups, which are negative, are buried in the membranes and only the choline groups, which are positive, show up. When the SUVs transit to the fluid phase, the membranes loosen up, the dipole heads flatten, diffuse laterally and scramble producing a zero zeta potential, see Fig. 3b. Let us emphasize this phenomenon: it is normally believed that zwitterionic lipids self-assembly into zwiterionic vesicles, but we have found that it is not true in our case; depending on the temperature, 20 nm vesicles can be positive or neutrally charged. In other words, the zwitterionic nature of the lipids is lost in rigid SUVs, but recovered when they are softer or in the fluid phase. It is intriguing that this phenomenon has been, as far as we know, unnoticeable.

The zeta potential of the polystyrene microparticles, on the contrary, is negative at all temperatures (∼−47 mV). Hence, SUVs and polystyrene colloids attract each other. The vesicles concentration, very low if they would have remain homogeneously distributed in the suspension (in fact, the volume fraction was 0.019), increases greatly to *ϕ* ∼ 0.58 in the vicinity of the microparticles, see ESI.† Such crowded environment or dense cloud of nanovesicles exerts a cage-like entrapment. Contrary to other asymmetric caging systems where small colloids (for instance, star polymers) entropically drive clustering of larger particles and in such scenario they vitrify, we found in our experiments that the dynamical arrest is produced by the smaller colloids onto larger ones. Moreover, instead of short-range attractions in the SALR interactions, long-range forces between small and large colloids are behind caging. Since in the cages there are only individual tracers, there is no rattling and therefore free diffusion is first observed. At larger lag times, subdiffusion shows up.

The origin of the large splitting of the MSD produced by the DMPC vesicles (see Fig. 2b) is now easily explained: below the transition temperatures the SUVs are rigid, above they are soft. In the fluid phase (*i.e.* when the SUVs are soft), the local viscosity of the surrounding medium decreases so the diffusion coefficient augments (indeed, soft deformable colloids always reduce the viscosity of a suspension^21,22^ and the diffusion coefficient is inversely proportional to it^23^). Considering the shape of the zeta potential curves of the DMPC vesicles (see Fig. 3b), we decided to measure the MSD of the tracers backwards (*i.e.* instead of heating the suspension from the gel to the fluid phase, as done in Fig. 2b, we cooled it from the fluid to the gel phase). For temperatures above the *T*_m_, the SUVs are quite neutral so they are not attracted to tracers and MSD are linear for all lag times, see non-geometric symbols in Fig. 2b. At lower temperatures, below *T*_m_, the vesicles turn positive, they rapidly surround tracers and sub-diffusion shows up again. This hysteresis confirms our claim that long-range attractive interactions are behind the dynamical arrest of large colloids. Should two hydrodynamic sizes (tracers alone and tracers with surrounding clouds of vesicles) exist, the diffusion coefficient would be just different without the emergence of a sub-diffusion regime.

We would like to comment that the MSD splittings produced by DMPC vesicles are greater than those produced by DPPC due to the length of the lipids. Both lipids have the same hydrophilic heads but different tails. The cohesion energy for DMPC is less than for DPPC, therefore, DMPC lipids have more freedom to move and deform in the vesicle surface. We can observe that this freedom is truely sensed in the DMPC pretransition peak (Fig. 3a), confirming previews results^24^ claiming that the pre-transition or rippling phase disappears at higher tension at the surface, see the DPPC profile in Fig. 3a. These results can be explained by the physical properties of each lipid (summarised in Table 1 in the ESI†), where we can see that the spontaneous curvature radio of DPPC is six times higher than DMPC, despite the fact that the only chemical difference is the smaller hydrocarbonated tail.

As above discussed, once the DMPC or DPPC vesicles are electrically charged with the negative lipids DMPA and DPPA, they cannot approach the polystyrene particles and neither sub-diffusion nor splitting show up (see insets in Fig. 2b and c).

**Fig. 4:**
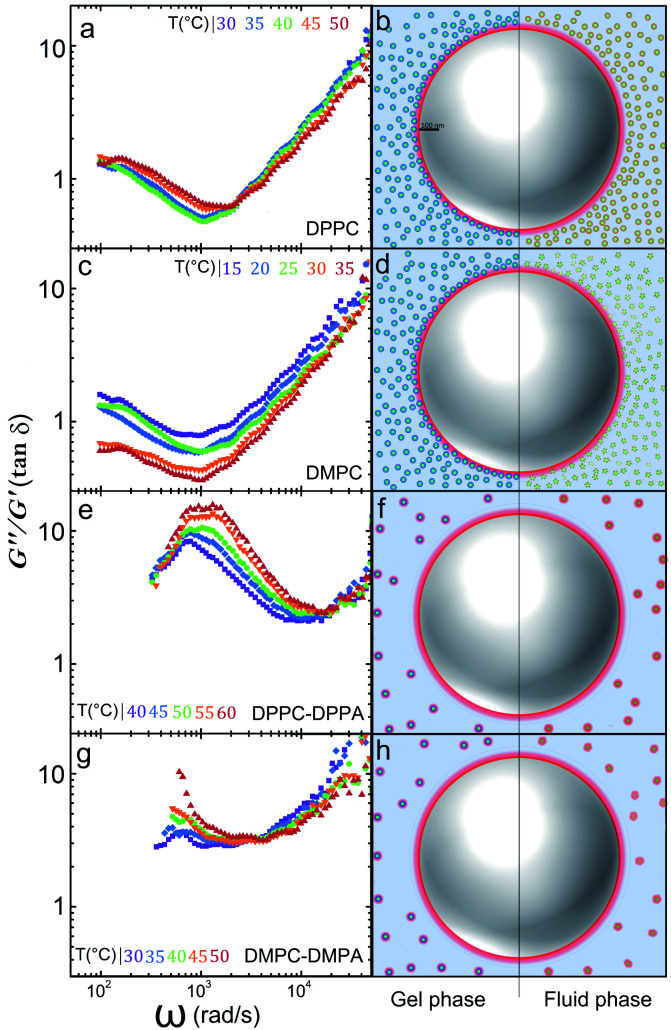
The *G*′′/*G*′ ratio (tan *δ*) for: (a) DPPC, (c) DMPC, (e) DPPC-DPPA, and (g) DMPC-DMPA. We schematize the different scenarios the particle may incur: (b) DPPC vesicles approach the polystyrene particles by long-rang electrostatic attraction; (d) DMPC vesicles approach the polystyrene particle by long-range electrostatic attraction. In both cases, the suspension is viscoelastic. In (f) and (h) negatively charged doped vesicles remain far away from the tracers and each other and the suspensions are purely viscous.

In a similar way the theory of dynamic light scattering, detailed in the ESI,† has been employed to evaluate the rheological moduli of colloidal suspensions,^25^ we use it here to scrutinize the microrheology of our colloidal mixtures and to summarize our findings described before. In Fig. 4 we show loss tangents, given by tan *δ* = *G*′′/*G*′, where *δ* is the phase difference between the stress and the strain, (plots A, C, E and G). If tan *δ* > 1 the sample is mostly a viscous Newtonian fluid, if tan *δ* < 1 is mostly elastic. Notoriously, by doping the vesicles with charged lipids, we can go from a viscoelastic regime to a purely viscous one.

We consider that our results, by their own, are novel and important, for individual microparticles or tracers are dynamically arrested by soft nanocolloids easily modified by temperature. However, if we ought to conceive a field where our present results may have some significance in biological systems, transport of proteins, lipids, neurotransmitters and RNAs is perhaps an indicated one. Recently, the function of small vesicles in the intercellular^26^ and extracellular communication medium,^27^ signalling, and regulation, have been of keen interest, as well as in neurotransmission,^28,29^ cancer^30^ and immunology.^31^ Charge is ubiquitous in biological systems so our results may help to shed some light in scenarios like the one here studied.

## 5 Conclusion

We have measured the mean square displacements (MSD) of tracer particles in a suspension of SUVs made of different lipid compositions at various temperatures. We found an interesting diffusion anomaly of the tracers in the presence of crowded environments of nanovesicles that hint to the existence of vitrification. Interestingly, no short-range entropic forces participate to form vitrified clusters of large particles, rather, long-range coulomb attractions are behind the observed nanovesicle glasses with single tracers trapped inside. Although a ballistic behavior due to rattling is not seen because tracers cannot rattle in a cage formed by very light nanoparticles, we do observe sub-diffusion of the tracers not seen previously in asymmetric glasses. Moreover, the MSD are sensitive to temperature, especially for shorter lipids. When the SUVs are doped with charged lipids, the diffusion becomes not only Newtonian but athermal. Our findings may contribute to the understanding of vesicle diffusion taking place in the neural presynaptic bouton, cancer metastasis and immunization. Indeed, small vesicles are surrounded by cells and proteins with diverse electrostatic distributions, where the vesicle stiffness and electrostatic charge could determine their function. Future work in our group will investigate the diffusion of nanovesicles in a dense protein medium.

## Conflicts of interest

There are no conflicts to declare.

## Supplementary Material

RA-011-D1RA04252A-s001
