# Supplementary material for: Nanovesicles drive a tunable dynamical arrest of microparticles
Source: RSC Adv. 2021 Jul 9;11(39):24190–5. doi: 10.1039/d1ra04252a (PMC9036661; doi:10.1039/d1ra04252a)
Supplement: RA-011-D1RA04252A-s001 [file RA-011-D1RA04252A-s001.pdf]

Electronic Supplementary Material (ESI) for RSC Advances.
This journal is © The Royal Society of Chemistry 2021

Nanovesicles Drive a Tunable Dynamical Arrest of Microparticles

Francisco Javier Guevara-Pantoja and J. C. Ruiz-Suárez*

CINVESTAV-Monterrey, PIIT, Autopista Nueva al Aeropuerto Km. 9.5, Apodaca, Nuevo León 66600, Mexico.

E-mail: jcrs.mty@gmail.com

Experimental data and characterization for all suspensions.

Table 1: Physical properties of lipids

Property	DPPC	DMPC
Spontaneous radius of curvature (Å)[1]	2465	392
Bilayer Thickness (nm)[2]	3.9	3.67
Area/lipid (Å^2)[3]	63	59.9
Bending constant[1]	15.8	12.2
Area compressibility (dyn/cm)[1]	230	210
Chemical structure	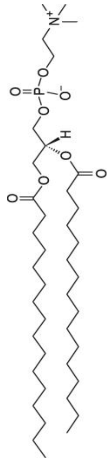	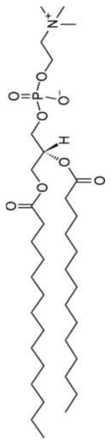

Dynamic Light Scattering

The MSD is a statistical measure of time-dependent particle displacements: $\langle \Delta r^2(\tau) \rangle = \langle [r(t+\tau) - r(t)]^2 \rangle$, where $r(t)$ and $r(t+\tau)$ are the positions of a single particle τ seconds apart. The angular brackets in the MSD indicate a time average on all such position pairs in a trajectory.

The mean squared displacements of the polystyrene tracer particles were obtained from the electric field autocorrelation function:

$$g_1(\tau) = g_1(0) exp[-\frac{q^2}{6} \langle \Delta r^2(\tau) \rangle], \quad (1)$$

where $g_1(0)$ is the correlation value at zero time (or intercept), q is the magnitude of the scattering vector, given by $q = (4\pi n/\lambda_o)(\sin(\theta/2))$, n is the refractive index of the solution, λ and θ the wavelength of the light and the scattering angle, respectively. Using the following equation:

$$\langle \Delta r^2(\tau) \rangle = \frac{6}{q^2} [log(g_1(0)) - log(g_1(\tau))], \quad (2)$$

we extract the MSD of the tracers.

Assuming the tracers are able to identify the vesicle suspension as a continuum, the viscoelastic modulus is obtained with a method based on a power law expansion of the MSD locally around $t = 1/\omega$. Then, by evaluating the Fourier transform of the MSD (Γ) and using Euler's equation results in the G' elastic (storage) and G" viscous (loss) modulus,[4] we get:

$$G'(\omega) = G^*(\omega) cos[\pi\alpha(\omega)/2],$$

and

$$G''(\omega) = G^*(\omega) sin[\pi\alpha(\omega)/2],$$

where $G^*(\omega)$ is given by:

$$|G^*(\omega)| = \frac{k_b T}{\pi a(\langle \Delta r^2(\frac{1}{\omega})\rangle)\Gamma\left[1+\alpha(\omega)\right]}. \tag{3}$$

In the above equation a is the diameter of the particle, T is the temperature, k_b is the Boltzmann constant, Γ denotes the gamma function which is a result of the Fourier transform of the power law behaviour of the MSD and

$$\alpha(\omega) = \left.\frac{d\ln\langle \Delta r^2(t)\rangle}{d\ln t}\right|_{t=1/w}. \tag{4}$$

Vesicles volume fraction

The volume fraction (ϕ) is a parameter broadly used to evaluate colloidal suspensions. In order to estimate this value in our experiments, we need to know how many vesicles are in the suspension. We calculate this value by:

$$N = \frac{N_t}{N_l}, \tag{5}$$

where N is the number of vesicles, N_t is the number of total lipids and N_l is the number of lipids of one vesicle. Using basic chemical and geometry equations we can obtain both numbers by:

$$N_t = (\frac{C}{MM_l})(N_a), \tag{6}$$

$$N_l = (\frac{A_e + A_i}{A_l}), \tag{7}$$

where C is the mass of lipids, MM_l is the molar mass of the lipid, N_a the Avogadro's number, A_e the external area of the vesicle, A_i the inner area of the vesicle and A_l the area needed for each lipid. Knowing that the surface area of a sphere is $A = 4\pi r_e^2$, where the external and inner radii relate as $r_i = r_e - t_l$, where t_l is the lipid bilayer thickness ≈ 4 nm, we can

simplify the equation to obtain the total number of vesicles as:

$$N = \frac{K}{r_e^2 - 4r_e + 8}, \tag{8}$$

where:

$$K = \frac{CNaA_l}{8\pi MM_l}, \tag{9}$$

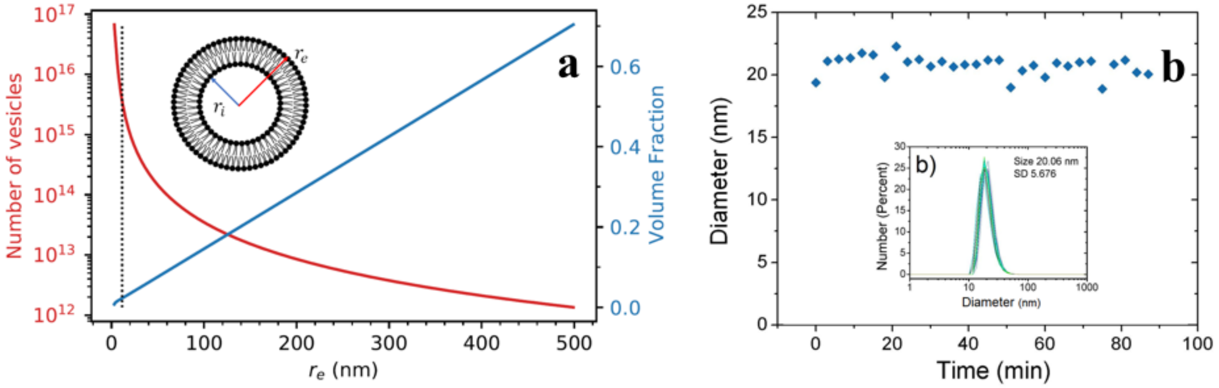

Figure 1: (a) Vesicle number and volume fraction as a function of vesicle size, dot line marks our experimental conditions. (b) DMPC Vesicle size evolution through 90 min, note that the size does not change during the DLS measurements.

Now, we can obtain the total volume of the vesicles and therefore the volume fraction by $\phi = \frac{\frac{4}{3}\pi r_e^3(N)}{V_t}$, where V_t is the total volume of the sample. Applying the information of the table 1, the experimental data for DMPC, and the concentration of 15 mg/ml, we can obtain N and ϕ as function of r_e, see Fig. 1a.

Knowing the number of vesicles and tracer particles (volume concentration divided by the volume of each particle $N_p = \frac{V_c}{V_p}$), we obtain for DMPC SUVs (at 15 mg/ml) that there are approximately 1030836 vesicles per tracer particle. In the limiting case where all nanovesicles are attracted to tracers, it is easy to calculate that within a ≈ 0.55 μm circlet around each tracer, the local volume fraction would be $\phi = 0.64$, the maximum possible value. Such crowded environment induces the virtual cage of tracers.

We corroborate that the vesicles size remains stable during the time of an experiment,

see Fig. 1b.

Finally, by visual inspection, we verify that there is no aggregation of tracer particles during the time of the experiments, see Fig 2.

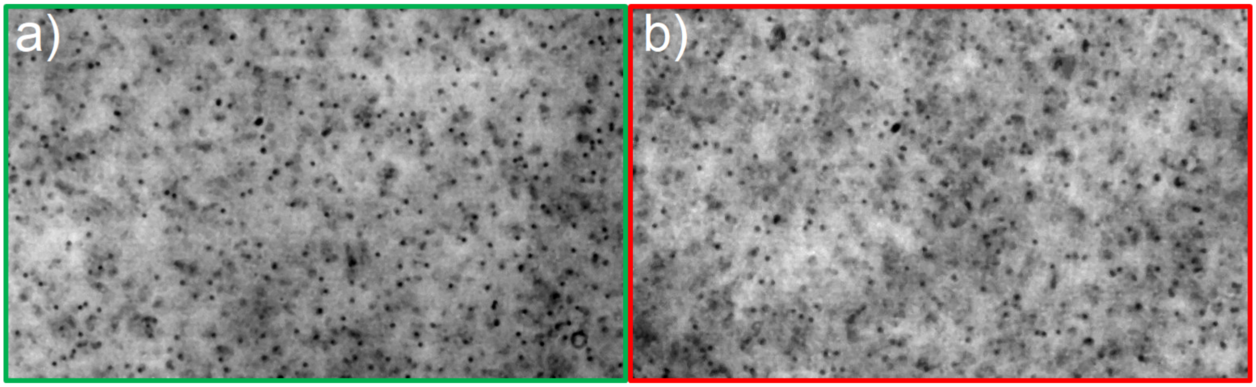

Figure 2: Representative picture of (a) before and (b) after the rheology experiment. Black dots are 0.994 μm tracer particles in the suspension of SUVs (which are no visible).

References

(1) Venable, R. M.; Brown, F. L.; Pastor, R. W. *Chemistry and Physics of Lipids* **2015**, *192*, 60–74.

(2) Kučerka, N.; Nagle, J. F.; Sachs, J. N.; Feller, S. E.; Pencer, J.; Jackson, A.; Katsaras, J. *Biophysical Journal* **2008**, *95*, 2356–2367.

(3) Kučerka, N.; Nieh, M. P.; Katsaras, J. *Biochimica et Biophysica Acta - Biomembranes* **2011**, *1808*, 2761–2771.

(4) Mason, T. G. *Rheologica Acta* **2000**, *39*, 371–378.
